# Supplementary material for: Executive dysfunction is associated with altered hippocampal-prefrontal functional connectivity in 3xTg Alzheimer’s model mice
Source: Commun Biol. 2025 Aug 6;8:1163. doi: 10.1038/s42003-025-08546-2 (PMC12329050; doi:10.1038/s42003-025-08546-2)
Supplement: Supplementary file 1 — Supplementary Information [file 42003_2025_8546_MOESM1_ESM.pdf]

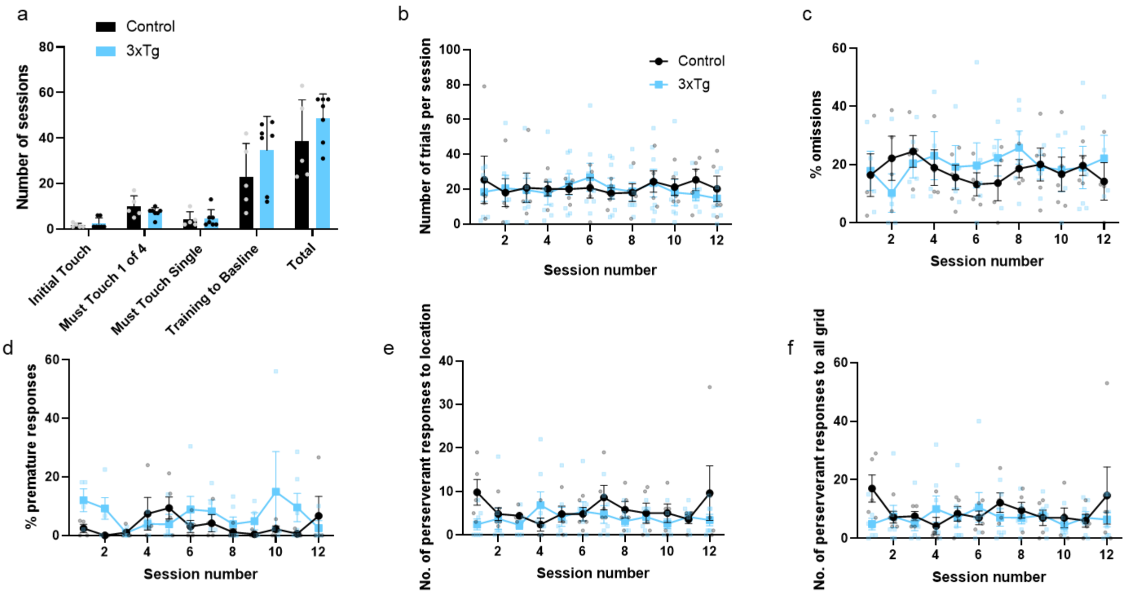

Supplementary figure 1: No significant differences in the sessions required to reach criterion, % omissions, % premature responses, or number of perseverant responses between 3xTg and control mice on the 4CGT. a Comparison between controls and 3xTgs in the number of sessions required to reach criterion at each training stage, and overall. **b** Comparison between controls and 3xTgs in the number of trials completed per session. **c-f** Comparisons between controls and 3xTgs in the **c** % omissions, **d** % premature responses, and number of perseverant responses to either **e** choice location or **f** all of grid, for each session. n= 5 controls and n= 7 3xTgs. Error bars indicate SEM.

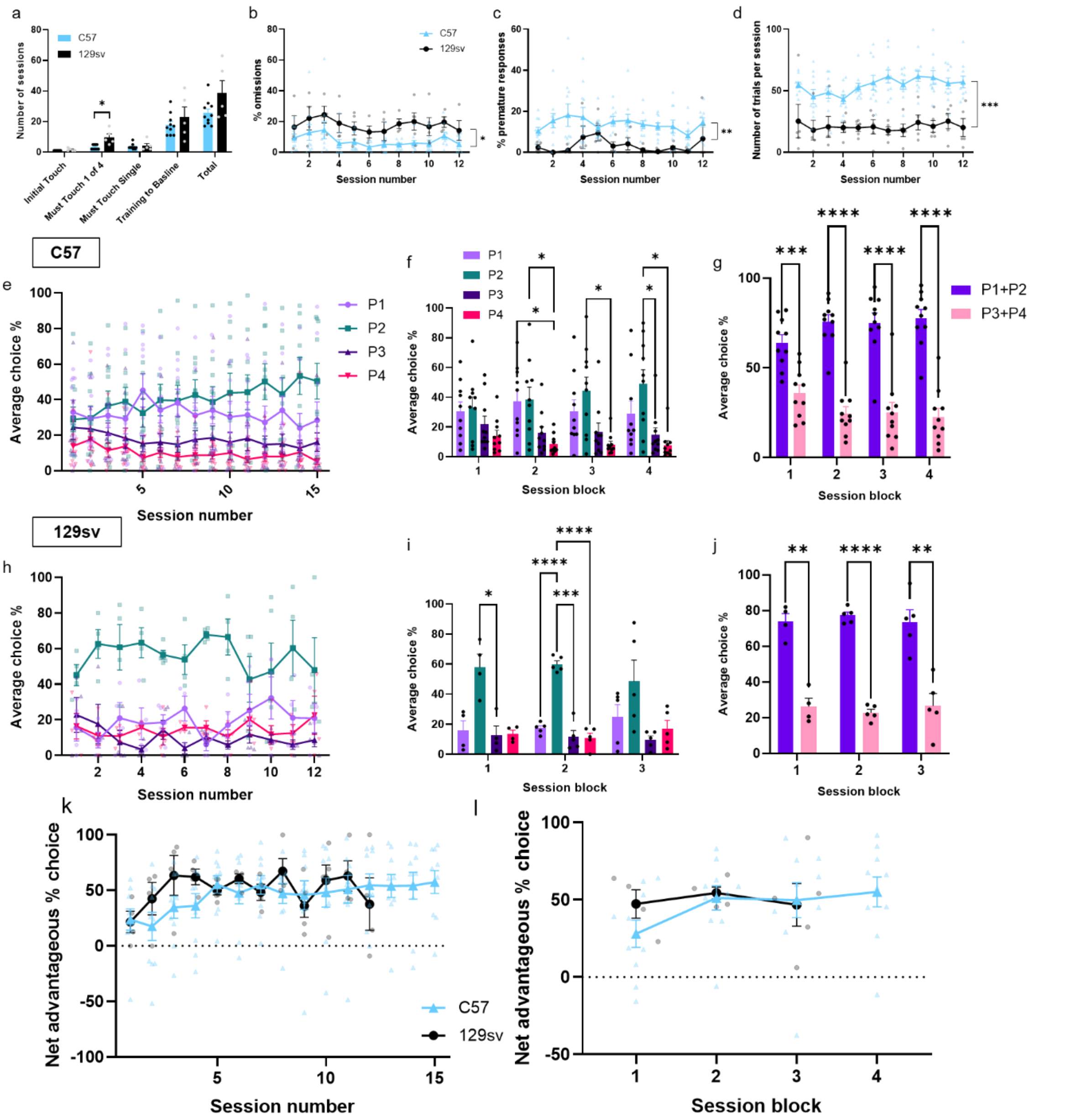

Supplementary figure 2: Performance of C57BL/6 (C57) and C57/129sv (129sv) control mice on the 4CGT. a Comparison of the number of sessions taken to reach criterion at each training stage, and overall, between C57 and 3xTg 129sv control mice **b-d** Comparison of the **b** % omissions, **c** % premature responses, and **d** number of trials per session, between C57 and 129sv mice. **(e)** Average choice % of C57 mice by session. **f** The same data as for **e**, but with sessions displayed in blocks of 4. **g** Average choice % of C57 mice for advantageous (P1+P2) vs disadvantageous (P3+P4) choices, by session block. **h** Average choice % of 129sv mice by session. **i** The same data as for **h**, but with sessions displayed in blocks of 4. **j** Average choice % of 129sv mice for advantageous (P1+P2) vs disadvantageous (P3+P4) options, by session block. **k** Net advantageous choice % by individual session. This was calculated by subtracting the average % of disadvantageous choices (P3+P4) from the average % of advantageous choices (P1+P2) selected by mice across the session. The more positive the value, the better the decision-making and task performance. **l** The same data as for **k**, but with sessions blocked. Both strains were able to implement an advantageous strategy and choose a higher % of advantageous than disadvantageous choices. n= 10 female C57 mice, n= 5 male 129sv mice. Error bars indicate SEM. *p<0.05, **p<0.01, ***p<0.001, ****p<0.0001.
