# Supplementary material for: Executive dysfunction is associated with altered hippocampal-prefrontal functional connectivity in 3xTg Alzheimer’s model mice
Source: Commun Biol. 2025 Aug 6;8:1163. doi: 10.1038/s42003-025-08546-2 (PMC12329050; doi:10.1038/s42003-025-08546-2)
Supplement: Supplementary file 4 — Reporting summary [file 42003_2025_8546_MOESM4_ESM.pdf]

nature portfolio

Corresponding author(s): Dr John Gigg

Last updated by author(s): Jul 3, 2025

Reporting Summary

Nature Portfolio wishes to improve the reproducibility of the work that we publish. This form provides structure for consistency and transparency in reporting. For further information on Nature Portfolio policies, see our Editorial Policies and the Editorial Policy Checklist.

Statistics

For all statistical analyses, confirm that the following items are present in the figure legend, table legend, main text, or Methods section.

n/a	Confirmed	
☐	☒	The exact sample size (n) for each experimental group/condition, given as a discrete number and unit of measurement
☐	☒	A statement on whether measurements were taken from distinct samples or whether the same sample was measured repeatedly
☐	☒	The statistical test(s) used AND whether they are one- or two-sided<br>*Only common tests should be described solely by name; describe more complex techniques in the Methods section.*
☐	☒	A description of all covariates tested
☐	☒	A description of any assumptions or corrections, such as tests of normality and adjustment for multiple comparisons
☐	☒	A full description of the statistical parameters including central tendency (e.g. means) or other basic estimates (e.g. regression coefficient) AND variation (e.g. standard deviation) or associated estimates of uncertainty (e.g. confidence intervals)
☐	☒	For null hypothesis testing, the test statistic (e.g. F, t, r) with confidence intervals, effect sizes, degrees of freedom and P value noted<br>*Give P values as exact values whenever suitable.*
☒	☐	For Bayesian analysis, information on the choice of priors and Markov chain Monte Carlo settings
☒	☐	For hierarchical and complex designs, identification of the appropriate level for tests and full reporting of outcomes
☒	☐	Estimates of effect sizes (e.g. Cohen's d, Pearson's r), indicating how they were calculated

Our web collection on statistics for biologists contains articles on many of the points above.

Software and code

Policy information about availability of computer code

Data collection	Behavioural data was collected using ABET II software (version 2. 16. 1, Campden Instruments Ltd, UK). Electrophysiological data was collected using Signal software (version 5; CED, UK).
Data analysis	Electrophysiological traces were averaged and responses and latencies measured using Signal software (version 5; CED, UK). Prism software (version 9.5.1, GraphPad UK) was used to analyse all data, and present behavioural and electrophysiological data.

For manuscripts utilizing custom algorithms or software that are central to the research but not yet described in published literature, software must be made available to editors and reviewers. We strongly encourage code deposition in a community repository (e.g. GitHub). See the Nature Portfolio guidelines for submitting code & software for further information.

Data

Policy information about availability of data

All manuscripts must include a data availability statement. This statement should provide the following information, where applicable:

- Accession codes, unique identifiers, or web links for publicly available datasets
- A description of any restrictions on data availability
- For clinical datasets or third party data, please ensure that the statement adheres to our policy

All data are available upon request to the corresponding author.

Research involving human participants, their data, or biological material

Policy information about studies with human participants or human data. See also policy information about sex, gender (identity/presentation), and sexual orientation and race, ethnicity and racism.

Reporting on sex and gender	n/a
Reporting on race, ethnicity, or other socially relevant groupings	n/a
Population characteristics	n/a
Recruitment	n/a
Ethics oversight	n/a

Note that full information on the approval of the study protocol must also be provided in the manuscript.

Field-specific reporting

Please select the one below that is the best fit for your research. If you are not sure, read the appropriate sections before making your selection.

☒ Life sciences ☐ Behavioural & social sciences ☐ Ecological, evolutionary & environmental sciences

For a reference copy of the document with all sections, see nature.com/documents/nr-reporting-summary-flat.pdf

Life sciences study design

All studies must disclose on these points even when the disclosure is negative.

Sample size	Sample size was determined based on animal availability and pilot data.
Data exclusions	For behavioural data, if a mouse completed less than 10 trials over a session, this individual performance was excluded from the grouped analysis. Sessions were excluded if less than n=4 mice from either genotype completed more than 10 trials over the session.<br>For electrophysiological data, traces were excluded from analysis if no local field response in the prefrontal cortex was observed following hippocampal stimulation.
Replication	No attempts made.
Randomization	Allocation was not random as mice had to be allocated to groups based on their genotype (control vs 3xTg).
Blinding	All experimental procedures and analyses were performed by G.C., and so blinding was not possible due to the necessity to individually prepare and handle the animals.

Reporting for specific materials, systems and methods

We require information from authors about some types of materials, experimental systems and methods used in many studies. Here, indicate whether each material, system or method listed is relevant to your study. If you are not sure if a list item applies to your research, read the appropriate section before selecting a response.

Materials & experimental systems

n/a	Involved in the study	
☒	☐	Antibodies
☒	☐	Eukaryotic cell lines
☒	☐	Palaeontology and archaeology
☐	☒	Animals and other organisms
☒	☐	Clinical data
☒	☐	Dual use research of concern
☒	☐	Plants

Methods

n/a	Involved in the study	
☒	☐	ChIP-seq
☒	☐	Flow cytometry
☒	☐	MRI-based neuroimaging

Animals and other research organisms

Policy information about studies involving animals; ARRIVE guidelines recommended for reporting animal research, and Sex and Gender in Research

Laboratory animals	This study used male triple transgenic (3xTgAD) mice carrying APPSWE, PS1M146V and TauP301L transgenes, and matched controls of the same background strain (C57/129sv) (Oddo et al., 2003). For supplementary data, C57BL/6 female mice were used.
Wild animals	This study did not involve wild animals.
Reporting on sex	Due to limitations on animal numbers, all experiments included in this study (except for supplementary figures from pilot studies) were conducted using exclusively male mice.
Field-collected samples	This study did not involve samples collected from the field.
Ethics oversight	All experimental procedures were performed under a Home Office UK project licence in accordance with the Animals (Scientific Procedures) Act 1986, and approval by the University of Manchester Animal Welfare and Ethical Review Body (AWERB).

Note that full information on the approval of the study protocol must also be provided in the manuscript.

Plants

Seed stocks	n/a
Novel plant genotypes	n/a
Authentication	n/a
